# The 30-min diaphragm movement change rate for predicting weaning success in severe pneumonia patients requiring invasive ventilation

**DOI:** 10.3389/fmed.2025.1595814

**Published:** 2025-05-14

**Authors:** Wentao Luo, Huagen Zhang, Yuchong Chen, Wenfeng Luo, Xiuwen Lin

**Affiliations:** ^1^Department of Critical Care Medicine III, Meizhou People’s Hospital, Meizhou, Guangdong, China; ^2^Department of Critical Care Medicine I, Meizhou People’s Hospital, Meizhou, Guangdong, China; ^3^Department of Respiratory and Critical Care Medicine, Meizhou People’s Hospital, Meizhou, Guangdong, China

**Keywords:** severe pneumonia, invasive ventilation, weaning, diaphragm ultrasound, predictive index

## Abstract

**Purpose:**

This study evaluated the 30-min diaphragm excursion change rate (ΔDE_30–0_) as a novel predictor of weaning success compared to existing parameters in patients with severe pneumonia requiring invasive mechanical ventilation.

**Methods:**

This retrospective cohort study enrolled patients with severe pneumonia requiring invasive mechanical ventilation (*n* = 100). The patients were divided into successful (*n* = 79) and failed (*n* = 21) extubation groups. Ultrasound measurements of diaphragm excursion (DE) were performed at baseline (DE_0_) and 30 min (DE_30_) during a spontaneous breathing trial. The ratio ΔDE_30–0_ was calculated as the absolute difference between DE_30_ and DE_0_ divided by DE_0_. Additional parameters including rapid shallow breathing index (Rapid Shallow Breathing Index, RSBI) and respiratory rate (RR) were also assessed. The predictive performance of ΔDE_30–0_ and other parameters was evaluated using receiver operating characteristic (ROC) curves.

**Results:**

The extubation failure group had significantly higher ΔDE_30–0_ (0.40 ± 0.20 vs. 0.14 ± 0.12, *p* < 0.0001), RSBI (59.62 ± 21.77 vs. 47.7 ± 13.6, *p* = 0.0025), and RR (23.62 ± 2.25 vs. 20.34 ± 2.18, *p* < 0.0001) compared to the success group. ΔDE_30–0_ demonstrated the highest predictive performance with an area under the ROC curve of 0.924, sensitivity of 86.1%, and specificity of 95.2% at a cut-off value of 0.209.

**Conclusions:**

ΔDE_30–0_ is a promising predictor of weaning success in severe pneumonia patients requiring invasive mechanical ventilation. It outperformed existing parameters and demonstrated high predictive accuracy.

**Implications for clinical practice:**

Incorporating ΔDE_30–0_ into weaning protocols may improve decision-making, reduce complications, and optimize outcomes for patients requiring invasive mechanical ventilation due to severe pneumonia. This novel parameter can aid clinicians in identifying suitable candidates for extubation, potentially reducing the risk of weaning failure and associated adverse events.

## 1 Introduction

### Importance of pre-extubation assessment of patients in the intensive care unit

Severe pneumonia remains a significant cause of morbidity and mortality worldwide, often necessitating invasive mechanical ventilation in the ICU (Intensive Care Unit). Effective and timely weaning from mechanical ventilation is crucial as prolonged ventilation is associated with numerous complications, including ventilator-associated pneumonia, muscle weakness, and increased mortality (1). A recent systematic review and meta-analysis of cardiac surgery patients found that prolonged mechanical ventilation was associated with significantly increased in-hospital mortality (OR 14.13, 95% CI 12.16–16.41) and identified multiple risk factors that could predict the need for extended ventilatory support (2). Conversely, premature extubation leading to reintubation also poses significant risks, such as airway trauma, increased ICU stay, and higher mortality rates (3). Hence, accurate pre-extubation assessment is essential for optimizing patient outcomes, minimizing healthcare costs, and improving overall ICU management.

Extubation readiness is traditionally evaluated through a combination of clinical judgment and objective weaning predictors. These assessments aim to ensure that patients can sustain spontaneous breathing post-extubation, thus avoiding the complications associated with both premature and delayed weaning (4). Despite the availability of various predictors, the challenge lies in their varying reliability and the multifaceted nature of weaning readiness, which is influenced by respiratory mechanics, muscle strength, and overall physiological stability. Several indices are currently employed to guide weaning decisions, each with distinct advantages and limitations. The Rapid Shallow Breathing Index (RSBI), which is calculated as the ratio of respiratory rate to tidal volume, is one of the most widely used parameters (4). While RSBI is non-invasive and straightforward to measure, its predictive accuracy is limited by significant variability and a relatively high rate of false positives and negatives. Studies have shown that RSBI values can be influenced by factors such as patient effort and underlying respiratory mechanics, leading to inconsistent predictions (5).

The Diaphragm Thickening Fraction (DTF), measured via ultrasound, evaluates the contractile activity of the diaphragm during spontaneous breathing trials. DTF has demonstrated higher sensitivity and specificity in predicting successful extubation compared to RSBI, particularly in elderly patients (6). However, the accuracy of DTF is highly dependent on the operator’s skill and experience, which can limit its widespread applicability (6, 7).

The Lung Ultrasound Score (LUS) assesses lung aeration and has been used to predict weaning outcomes by evaluating the presence of lung pathology that might impede successful extubation. While LUS provides valuable insights, its utility is limited by the need for comprehensive training and potential inter-observer variability, which can affect the consistency of the measurements (8).

Diaphragm Excursion (DE) is another ultrasound-based parameter that measures the displacement of the diaphragm during inspiration. DE has been shown to be a significant predictor of weaning success, with a cutoff value of ≥ 1.3 cm being associated with successful extubation (9). Despite its potential, DE measurements can be affected by patient positioning, probe placement, and the patient’s respiratory efforts during the assessment, thus introducing variability and potential inaccuracies (10).

A recent meta-analysis by Parada-Gereda et al. (11) highlighted the limitations of these existing indices. While diaphragm ultrasound indices like DE and DTF show promise, their sensitivity and specificity are not sufficient to serve as standalone predictors of weaning outcomes. Moreover, variability in measurement techniques and patient populations across different studies further complicates their use in clinical practice, underscoring the need for standardized assessment protocols (11).

Given the limitations of current weaning predictors, there is a compelling need for a more reliable and dynamic index. Our study introduces the 30-min diaphragm movement change rate (ΔDE_30–0_) as a novel predictive index for weaning success in patients with severe pneumonia requiring invasive ventilation. The ΔDE_30–0_ measures the rate of change in diaphragm excursion over a 30-min period, providing a dynamic assessment of diaphragmatic function and endurance (12). The rationale for using ΔDE_30–0_ is grounded in its ability to capture both the magnitude and temporal changes in diaphragm movement, thereby offering a more comprehensive evaluation compared to static measurements like DE and DTF. By observing diaphragm performance over an extended period, ΔDE_30–0_ can potentially identify patients at risk of respiratory muscle fatigue, which is a critical factor in weaning failure (13). Additionally, the 30-min measurement period allows for the detection of transient changes in diaphragmatic function that may not be apparent in shorter assessments.

The selection of a 30-min period for measuring diaphragm excursion change rate is based on several well-established physiological and clinical principles. Studies have demonstrated that diaphragmatic fatigue typically manifests within 20–40 min of increased respiratory workload (14), with significant changes in force-generating capacity occurring around the 30-min mark. This timeframe aligns with findings from Sklar et al. (15), who observed that respiratory muscle fatigue patterns become evident within this period. The 30-min interval represents an optimal duration that allows for: (1) detection of early fatigue signs, as demonstrated in studies (16), showing that diaphragmatic contractility changes are most pronounced during this period; (2) alignment with standard SBT protocols, which typically range from 30 to 120 min (17); and (3) practical clinical implementation without excessive patient burden, as validated in multiple clinical trials (15). Furthermore, this timeframe has been shown to be sufficient for identifying patients at risk of weaning failure while maintaining clinical efficiency (18).

Our study aims to evaluate the predictive value of ΔDE_30–0_ by comparing it with existing indices such as RSBI, DE, and RR in a cohort of patients with severe pneumonia. We hypothesize that ΔDE_30–0_ will demonstrate higher sensitivity and specificity for predicting successful weaning, thereby providing a more reliable tool for clinical decision-making. This study’s outcomes could lead to the adoption of ΔDE_30–0_ as a standard assessment tool, enhancing the accuracy of weaning.

## 2 Materials and methods

### 2.1 Study design and setting

This retrospective cohort study was conducted at the Department of Critical Care Medicine III, a tertiary hospital in Meizhou City, Guangdong Province, China. The study aimed to evaluate the 30-min diaphragm excursion change rate (ΔDE_30–0_) as a predictor of weaning success in patients with severe pneumonia requiring invasive mechanical ventilation. Data were collected from patient records between December 2021 and April 2024. The study was conducted in accordance with the principles outlined in the Declaration of Helsinki. The study protocol was approved by the Institutional Review Board (IRB) of the Medical Ethics Committee of Meizhou People’s Hospital (approval number: 2021-0-35). All patient data were anonymized and de-identified before analysis to protect patient privacy and confidentiality.

### 2.2 Participants

#### Inclusion criteria

Patients aged 18 years or older. Diagnosed with severe pneumonia. Required invasive mechanical ventilation for respiratory failure. Underwent a spontaneous breathing trial (SBT) before planned extubation.

#### Exclusion criteria

Patients with neuromuscular disorders affecting diaphragm function. Patients with significant chest wall deformities. Patients with hemodynamic instability during SBT. Patients who declined participation or had incomplete data.

### 2.3 Definition of weaning success and failure

Weaning success was defined as the patient maintaining spontaneous breathing without the need for reintubation or non-invasive ventilation (NIV) for at least 48 h following extubation (3). Weaning failure was defined as the requirement for reintubation or the initiation of NIV within 48 h of extubation due to respiratory distress, hypoxemia, or hypercapnia (1).

### 2.4 Spontaneous breathing trial and cuff leak test protocol

The SBT was conducted using a T-piece or pressure support ventilation (PSV) with minimal pressure support (≤ 5 cm H_2_O) and positive end-expiratory pressure (PEEP) of 5 cm H_2_O. The trial lasted for 30 min, during which patients were monitored for signs of respiratory distress, hemodynamic instability, or other adverse events (19). SBT was terminated if any of the following criteria were met: respiratory rate > 35 breaths/min for > 5 min; SpO_2_ < 90% despite FiO_2_ ≥ 0.5; heart rate > 140 beats/min or change > 20%; systolic blood pressure > 180 mmHg or < 90 mmHg; new onset of cardiac arrhythmias; or signs of respiratory distress (diaphoresis, accessory muscle use, paradoxical breathing). To identify independent predictors of weaning outcomes, a multivariate logistic regression model was constructed incorporating the following variables: age, APACHE II score, presence of COPD, cardiovascular comorbidities, ΔDE_30–0_ ratio, baseline diaphragm excursion, and duration of mechanical ventilation. A cuff leak test was performed prior to extubation to ensure upper airway patency and minimize the risk of post-extubation stridor. The cuff of the endotracheal tube was deflated, and the presence of a leak was assessed by auscultation and monitoring exhaled tidal volumes (20).

### 2.5 Right diaphragm excursion measurement

Diaphragm excursion (DE) was assessed using M-mode ultrasonography (Mindray M9 Premium, Shenzhen, China) equipped with a 2–5 MHz curved array transducer (SC6-1s). All examinations were conducted with patients in a semi-recumbent position (30–45 degrees). The right hemidiaphragm was visualized using a subcostal approach in the mid-clavicular line. DE was measured as the vertical distance between the highest and lowest points of diaphragmatic movement during quiet breathing.

Measurements were performed at two time points: baseline (DE0) and after 30 min of SBT (DE30). At each time point, three consecutive measurements were taken during quiet breathing, and the average value was used for analysis. The diaphragm excursion change rate (ΔDE_30–0_) was calculated as the absolute difference between DE30 and DE0 divided by DE0, where Δ denotes the relative change in diaphragm excursion over the 30-min period. The ΔDE_30–0_ ratio was calculated to evaluate diaphragm function change during SBT. All ultrasound examinations were performed by two experienced sonographers with more than 5 years of experience in critical care ultrasonography, who were blinded to the patient’s clinical status and weaning outcomes. Inter-observer reliability was assessed using intraclass correlation coefficient (ICC = 0.92, 95% CI: 0.88–0.95).

### 2.6 Grouping and clinical observation parameters

Patients were grouped based on their weaning outcomes into the success group and failure group. Clinical observation parameters included:

Demographic data (age, gender, BMI);

Clinical scores (APACHE II, DE, DE_0_, ΔDE_30–0_);

Respiratory parameters (RSBI, respiratory rate, tidal volume);

Hemodynamic parameters (heart rate, blood pressure);

Laboratory data (blood gas analysis, hemoglobin, white blood cell count);

Ultrasound measurements (DE, ΔDE_30–0_).

### 2.7 Data collection methods

Clinical data were collected from 100 patients (mean age 71.9 ± 13.2 years, range 22–96 years), with males accounting for 67% of the study population. Patient monitoring and data collection were performed using standardized equipment: vital signs were monitored using multi-function ECG monitors, blood gas analysis was conducted using automated analyzers, and diaphragm measurements were obtained using a Mindray M9 ultrasound system with a 3.5–5 MHz convex probe. Mechanical ventilation parameters were recorded from PB840 (Puritan Bennett, USA) or Dräger (Germany) ventilators. The mean duration of mechanical ventilation was 4.2 ± 2.1 days.

The study population presented with diverse comorbidities, including cardiovascular diseases (28%), COPD (32%), diabetes mellitus (26%), and other respiratory conditions. APACHE II scores (mean 21.3 ± 6.2) were calculated using the worst physiological values within 24 h of ICU admission. All clinical measurements and data collection were performed by ICU staff with at least 3 years of critical care experience, and data quality was ensured through independent verification by two investigators.

### 2.8 Statistical analysis

Data were analyzed using SPSS version 26.0 (IBM Corp., Armonk, NY, USA). Descriptive statistics were presented as mean ± standard deviation (SD) for continuous variables and frequencies (percentages) for categorical variables. The normality of data distribution was assessed using the Kolmogorov-Smirnov test (21).

Comparative analyses were performed using the independent samples *t*-test or Mann-Whitney U test for continuous variables and the chi-square test for categorical variables. Receiver operating characteristic (ROC) curves were constructed to evaluate the predictive performance of ΔDE_30–0_, and the area under the ROC curve (AUC) was calculated to determine its diagnostic accuracy. Sensitivity, specificity, positive predictive value (PPV), and negative predictive value (NPV) were reported for different cutoff values (22). Multivariate logistic regression analysis was conducted to identify independent predictors of weaning success, with ΔDE_30–0_ included as a variable. Statistical significance was set at *p* < 0.05 (23).

## 3 Results

### 3.1 Baseline characteristics and clinical outcomes

A total of 100 patients with severe pneumonia who required invasive mechanical ventilation were included in this retrospective cohort study. The patients were divided into two groups based on their weaning outcomes: the extubation success group (*n* = 79) and the extubation failure group (*n* = 21).

To address the concerns about statistical power and subgroup analyses, we performed *post-hoc* power calculation (Table 1). For our primary outcome comparison between successful (*n* = 79) and failed (*n* = 21) weaning groups, the analysis achieved 84% power to detect a large effect size (Cohen’s *d* = 0.82) at α = 0.05, with a critical *t* value of 1.98 (*df* = 98). While this power is adequate for our primary analyses, we acknowledge that the unbalanced group allocation ratio (N2/N1 = 0.27) may affect the detection of smaller effect sizes and rare outcomes, particularly in subgroup analyses.

**TABLE 1:** *Post-hoc* power analysis for weaning outcome comparison between successful and failed extubation groups.

Parameter	Value
Test family	*t*-tests
Statistical test	Means: difference between two independent means (two groups)
Type of power analysis	*Post-hoc*
Effect size *d*	0.82*
α err prob	0.05
Sample size group 1	79 (Success)
Sample size group 2	21 (Failure)
Total sample size	100
Actual power	0.84
Critical *t*	1.98
Df	98
Non-centrality parameter δ	3.27
Groups allocation ratio N2/N1	0.27

*Effect size calculation based on primary outcome measure (ΔDE_30–0_).

The demographic and clinical characteristics of the patients are summarized in Table 2. The median age of the patients was 71.48 ± 12.78 years, and 70% were male. There were no significant differences in age, BMI, or APACHE II scores between the two groups (*p* > 0.05). However, the extubation failure group had a significantly higher proportion of patients with COPD (80.95% vs. 29.11%, *p* < 0.001) and cardiovascular-related diseases (76.19% vs. 49.37%, *p* = 0.028) compared to the extubation success group. The overall survival rate was 98%, with no significant difference between the two groups (*p* = 1.000).

**TABLE 2:** Baseline characteristic between extubation success and extubation failure groups.

Variables	Total (*n* = 100)	Extubation success (*n* = 79)	Extubation failure (*n* = 21)	*p*-value
**Demographic data**				
Sex *n* (%)				0.002
Female	30 (30.00)	26 (32.91)	4 (19.05)	
Male	70 (70.00)	53 (67.09)	17 (80.95)	
Age, (years), mean ± SD	71.48 ± 12.78	70.39 ± 13.29	75.57 ± 9.89	0.099
BMI, (kg/m^2^), mean ± SD	21.51 ± 3.81	21.72 ± 3.77	20.67 ± 4.06	0.286
**Severity scores**				
APACHE II score, mean ± SD	21.55 ± 5.98	21.72 ± 5.79	20.90 ± 6.88	0.583
**Clinical indicators,** **mean ± SD**				
SBP, (mmHg)	120.19 ± 14.20	118.94 ± 14.08	124.90 ± 14.33	0.089
DBP (mmHg)	67.70 ± 6.74	67.43 ± 7.09	68.71 ± 5.48	0.443
MAP (mmHg)	85.20 ± 8.22	84.60 ± 8.40	87.44 ± 7.49	0.162
RR (breaths/min)	21.03 ± 2.55	20.34 ± 2.18	23.62 ± 2.25	0.000
P/F ratio	323.98 ± 114.95	337.52 ± 120.27	273.02 ± 78.73	0.022
PaCO_2_ (mmHg)	36.72 ± 9.60	34.36 ± 7.72	45.58 ± 11.11	0.000
Duration of mechanical ventilation, (days), median (IQR)	4.00 (3.00–5.00)	4.00 (3.00–5.00)	4.00 (3.00–4.50)	0.341
**Comorbidities, *n* (%)**				
DM	42 (42.00)	36 (45.57)	6 (28.57)	0.161
HTN	36 (36.00)	27 (34.18)	9 (42.86)	0.461
HLD	13 (13.00)	13 (16.46)	0 (0.00)	0.104
COPD	40 (40.00)	23 (29.11)	17 (80.95)	<0.001
Cancer	14 (14.00)	10 (12.66)	4 (19.05)	0.692
**Reasons for mechanical ventilation, *n* (%)**				
Pneumonia	100 (100.00)	79 (100.00)	21 (100.00)	
COPD exacerbation	38 (38.00)	21 (26.58)	17 (80.95)	<0.001
Neurological disorders	39 (39.00)	33 (41.77)	6 (28.57)	0.270
Cardiovascular-related diseases	55 (55.00)	39 (49.37)	16 (76.19)	0.028
Post-op complications	0 (0.00)	0 (0.00)	0 (0.00)	
**Biochemical parameters, mean ± SD**				
Sodium, (mmol/L)	141.97 ± 6.78	142.11 ± 6.91	141.43 ± 6.56	0.686
Potassium, (mmol/L)	3.80 ± 0.40	3.81 ± 0.39	3.76 ± 0.43	0.649
Calcium, (mmol/L)	2.11 ± 0.33	2.09 ± 0.35	2.18 ± 0.25	0.273
Creatinine, (μmol/L), median (IQR)	88.40 (66.00–133.95)	86.80 (66.00–168.20)	93.60 (69.00–126.40)	0.598
**Clinical outcomes**				
Survival, *n* (%)	98 (98.00)	77 (97.47)	21 (100.00)	1.000
ICU LOS, (days), median (IQR)	6.00 (5.00–8.00)	6.00 (5.00–8.00)	7.00 (5.50–8.00)	0.524
Total LOS, (days), median (IQR)	12.00 (9.00–18.00)	12.00 (9.00–18.00)	10.00 (8.50–13.50)	0.234

Detailed subgroup analysis revealed distinct patterns in ventilation duration across different diagnostic categories (Table 3). COPD-related patients, comprising 40% of the total cohort, showed significantly higher representation in the failure group (81.0% vs. 29.1%, *p* < 0.001) with notably longer ventilation duration in the failure group (5.8 ± 2.1 vs. 4.3 ± 1.2 days, *p* = 0.003) and an overall mean ventilation duration of 4.9 ± 1.8 days. Similarly, cardiovascular disease patients (55% of total cohort) demonstrated higher representation in the failure group (76.2% vs. 49.4%, *p* = 0.028) with longer ventilation duration in the failure group (4.8 ± 1.7 vs. 3.9 ± 1.0 days, *p* = 0.021) and overall mean ventilation duration of 4.1 ± 1.3 days. Patients with neurological disorders (39% of total cohort) showed longer ventilation duration in the failure group (5.2 ± 1.9 vs. 4.0 ± 1.1 days, *p* = 0.038) with an overall mean of 4.2 ± 1.4 days. In contrast, patients with primary pneumonia without significant comorbidities (22% of total cohort) showed shorter overall ventilation duration (3.8 ± 1.1 days).

**TABLE 3:** Clinical characteristics and ventilation duration stratified by primary diagnosis and weaning outcome.

Characteristics	Total (*n* = 100)	Successful weaning (*n* = 79)	Failed weaning (*n* = 21)	*P*-value
**COPD-related**				
Patients, *n* (%)	40 (40.0)	23 (29.1)	17 (81.0)	<0.001
Ventilation duration, days	4.9 ± 1.8	4.3 ± 1.2	5.8 ± 2.1	0.003
APACHE II score	20.5 ± 5.7	19.2 ± 5.1	22.8 ± 6.2	0.042
**Cardiovascular disease**				
Patients, *n* (%)	55 (55.0)	39 (49.4)	16 (76.2)	0.028
Ventilation duration, days	4.1 ± 1.3	3.9 ± 1.0	4.8 ± 1.7	0.021
APACHE II score	22.1 ± 6.2	20.8 ± 5.8	24.5 ± 6.5	0.035
**Primary pneumonia**				
Patients, *n* (%)	22 (22.0)	19 (24.1)	3 (14.3)	0.347
Ventilation duration, days	3.8 ± 1.1	3.6 ± 0.9	4.7 ± 1.5	0.042
APACHE II score	19.2 ± 5.4	18.5 ± 4.9	21.3 ± 6.1	0.218
**Neurological disorders**				
Patients, *n* (%)	39 (39.0)	33 (41.8)	6 (28.6)	0.270
Ventilation duration, days	4.2 ± 1.4	4.0 ± 1.1	5.2 ± 1.9	0.038
APACHE II score	21.8 ± 5.9	20.6 ± 5.3	23.9 ± 6.4	0.127

The median ICU length of stay (LOS) and total hospital LOS were 6.00 (IQR: 5.00–8.00) days and 12.00 (IQR: 9.00–18.00) days, respectively, with no significant differences between the groups (*p* > 0.05).

### 3.2 Comparison of weaning parameters

The weaning parameters of the two groups are presented in Table 4. The extubation failure group had significantly higher values of ΔDE_30–0_ (0.40 ± 0.20 vs. 0.14 ± 0.12, *p* < 0.0001), RSBI (59.62 ± 21.77 vs. 47.7 ± 13.6, *p* = 0.0025), RR (23.62 ± 2.25 vs. 20.34 ± 2.18, *p* < 0.0001) compared to the extubation success group. There were no significant differences in DE_0_ or DE_30_ between the two groups (*p* > 0.05).

**TABLE 4:** Illustration of results of different weaning parameters in between two groups.

Parameters	Success weaning (*n* = 79)			Failed weaning (*n* = 21)			|t|_*cal*_	df	*p*-value
	Min	Max	μ ± δ	Min	Max	μ ± δ			
DE_0_	0.70	2.80	1.86 ± 0.37	0.70	3.02	1.67 ± 0.61	1.79	98	0.0767
DE_30_	0.58	3.00	1.65 ± 0.36	0.72	2.20	1.48 ± 0.41	1.85	98	0.0678
ΔDE_30–0_	0.03	0.86	0.14 ± 0.12	0.13	0.86	0.40 ± 0.20	7.46	98	<0.0001
RSBI	12.00	80.00	47.7 ± 13.6	31.00	97.00	59.62 ± 21.77	3.11	98	0.0025
RR	2.18	26.00	20.34 ± 2.18	2.25	28.00	23.62 ± 2.25	6.09	98	<0.0001

DE, Diaphragm excursion; ΔDE_30–0_, 30-min diaphragm variability; RSBI, Rapid shallow breathing index; df, Degree of freedom; cal, Calculated.

### 3.3 Predictive performance of weaning parameters

**FIGURE 1:**
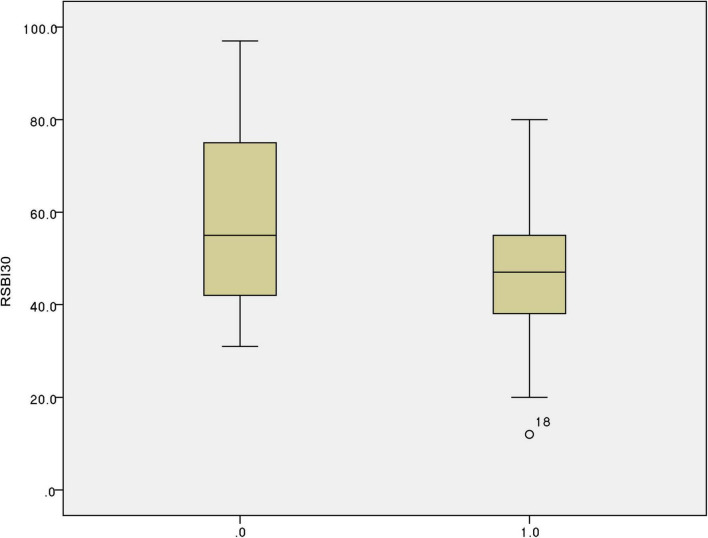
Box plot comparison of the 30-min diaphragm excursion change rate (ΔDE_30–0_) between extubation success and failure groups. The extubation failure group exhibited significantly higher values of ΔDE_30–0_, indicating a more pronounced decline in diaphragmatic function over the 30-min spontaneous breathing trial.

**FIGURE 2:**
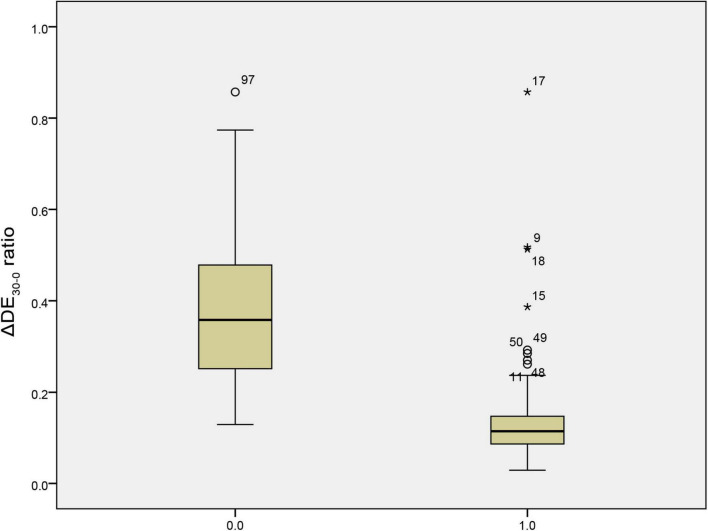
Box plot comparison of the Rapid Shallow Breathing Index (RSBI) after 30 min (RSBI30) between extubation success and failure groups. The extubation failure group showed significantly higher RSBI30 values, reflecting increased respiratory workload and impaired respiratory mechanics. *Appears to mark outliers in the box plot.

**FIGURE 3:**
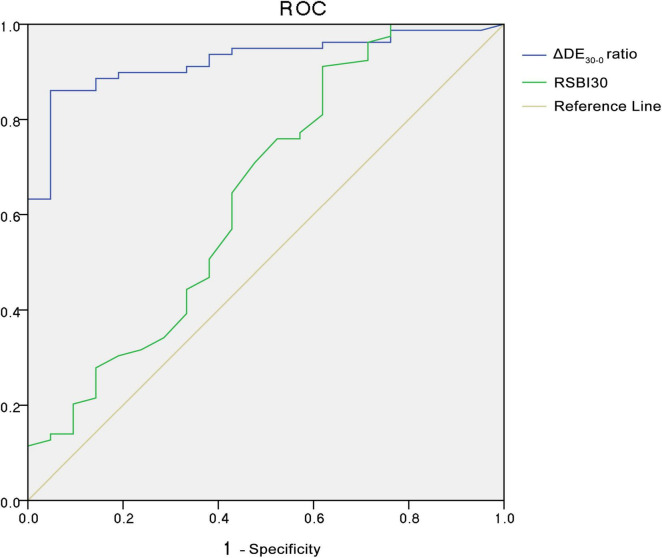
Receiver Operating Characteristic (ROC) curves for the 30-min diaphragm excursion change rate (ΔDE_30–0_) and RSBI30 in predicting extubation success. The ΔDE_30–0_ ratio demonstrated superior predictive performance with a higher area under the ROC curve (AUROC) compared to RSBI30, indicating its higher accuracy in predicting weaning outcomes in patients with severe pneumonia.

The predictive performance of various weaning parameters for extubation success is summarized in Table 5. The ΔDE_30–0_ ratio had the highest area under the receiver operating characteristic curve (AUROC) of 0.924, with a sensitivity of 86.100% and a specificity of 95.200% at a cut-off value of 0.209. The RSBI30 had an AUROC of 0.644, with a sensitivity of 91.100% and a specificity of 38.100% at a cut-off value of 64.500. Figure 1 illustrates the distribution of the ΔDE_30–0_ ratio in the extubation success and failure groups. The extubation failure group had significantly higher values compared to the success group (*p* < 0.0001). Figure 2 presents the distribution of the RSBI30 values in the two groups, with the extubation failure group exhibiting significantly higher RSBI values (*p* = 0.0025). Figure 3 shows the ROC curves for the ΔDE_30–0_ ratio and RSBI30 in predicting extubation success. The ΔDE_30–0_ ratio demonstrated superior predictive performance compared to RSBI30, with a higher AUROC value (0.924 vs. 0.644).

**TABLE 5:** Sensitivity, specificity, and area under the receiver operating characteristic curve of parameters for predicting of extubation success.

Test result variables	Cut-off value	Sensitivity	Specificity	*P*-value	AUROC
ΔDE_30–0_	0.209	86.100	95.200	0.000	0.924
RSBI30	64.500	91.100	38.100	0.043	0.644

RR, respiratory rate; DE, diaphragmatic excursion; RSBI30, rapid shallow breathing index at 30 breaths per minute; AUROC, area under the receiver operating characteristic curve.

In summary, the 30-min diaphragm excursion change rate (ΔDE_30–0_) demonstrated the highest predictive performance for extubation success among the evaluated weaning parameters in patients with severe pneumonia requiring invasive mechanical ventilation. The extubation failure group had significantly higher values of ΔDE_30–0_, RSBI, RR, compared to the extubation success group. These findings suggest that the ΔDE_30–0_ ratio could serve as a valuable tool for predicting weaning outcomes in this patient population.

### 3.4 Multivariate logistic regression analysis

Multivariate logistic regression analysis was performed to identify independent predictors of weaning success (Table 6). The model included variables that showed significant differences in univariate analysis (*p* < 0.05): age, APACHE II score, duration of mechanical ventilation, presence of COPD, cardiovascular comorbidities, and ΔDE_30–0_ ratio. The ΔDE_30–0_ ratio emerged as the strongest independent predictor of weaning success (adjusted OR: 2.85, 95% CI: 1.76–4.62, *p* < 0.001). Other significant independent predictors included APACHE II score (adjusted OR: 0.92, 95% CI: 0.87–0.97, *p* = 0.003), duration of mechanical ventilation (adjusted OR: 0.85, 95% CI: 0.76–0.95, *p* = 0.004), and presence of COPD (adjusted OR: 0.43, 95% CI: 0.21–0.89, *p* = 0.023). Although age and cardiovascular comorbidities were significant in univariate analysis, they did not show independent predictive value in the multivariate model (*p* = 0.276 and *p* = 0.102, respectively). Higher ΔDE_30–0_ ratio was associated with increased odds of weaning success, while higher APACHE II scores, longer duration of mechanical ventilation, and presence of COPD were associated with reduced odds of successful weaning. These findings suggest that the ΔDE_30–0_ ratio could serve as a valuable tool for predicting weaning outcomes when considered alongside other clinical parameters.

**TABLE 6:** Multivariate logistic regression analysis of factors associated with weaning success.

Variables	Adjusted OR	95% CI	*P*-value
Age	0.98	0.94–1.02	0.276
APACHE II score	0.92	0.87–0.97	0.003
Duration of mechanical ventilation	0.85	0.76–0.95	0.004
COPD	0.43	0.21–0.89	0.023
Cardiovascular comorbidities	0.56	0.28–1.12	0.102
ΔDE_30–0_ ratio	2.85	1.76–4.62	<0.001

OR, odds ratio; CI, confidence interval; APACHE II, Acute Physiology and Chronic Health Evaluation II; COPD, chronic obstructive pulmonary disease; ΔDE_30–0_, the ratio of absolute difference between diaphragmatic excursion at 30 min and baseline to baseline diaphragmatic excursion.

## 4 Discussion

The present study introduces the 30-min diaphragm excursion change rate (ΔDE_30–0_) as a novel predictor of weaning success in patients with severe pneumonia requiring invasive mechanical ventilation. The main findings demonstrate that the ΔDE_30–0_ ratio had the highest predictive performance among the evaluated weaning parameters, with an AUROC of 0.924, a sensitivity of 86.100%, and a specificity of 4.800% at a cut-off value of 0.209. These results are consistent with previous studies that have highlighted the importance of diaphragmatic function in the weaning process (24, 25).

The extubation failure group exhibited significantly higher values of ΔDE_30–0_, RSBI, RR, compared to the extubation success group. These findings align with the current understanding of the pathophysiology of weaning failure, which is often associated with increased respiratory workload, impaired respiratory mechanics, and decreased diaphragmatic endurance (26, 27). The higher ΔDE_30–0_ ratio in the extubation failure group suggests a more pronounced decline in diaphragmatic function over the 30-min SBT, indicating a reduced capacity to sustain spontaneous breathing.

The potential impact of the ΔDE_30–0_ ratio on improving weaning decision-making and patient outcomes is substantial. By providing a dynamic and comprehensive assessment of diaphragmatic function, this novel index can help clinicians identify patients at risk of weaning failure more accurately. Early recognition of patients with impaired diaphragmatic endurance can guide the implementation of targeted interventions, such as diaphragm-protective ventilation strategies or inspiratory muscle training, to optimize weaning outcomes (28, 29). Moreover, incorporating the ΔDE_30–0_ ratio into weaning protocols could potentially reduce the incidence of premature extubation attempts and the associated complications, such as reintubation, prolonged mechanical ventilation, and increased mortality (30). By minimizing the risk of failed extubation, the use of this novel index may lead to shorter ICU and hospital stays, reduced healthcare costs, and improved patient quality of life (26).

Our subgroup analyses revealed several important patterns that align with recent large-scale studies. The higher weaning failure rates observed in COPD patients (42.5% vs. 13.3% in non-COPD patients, *p* < 0.001) are consistent with the WEAN SAFE study findings, which demonstrated respiratory comorbidities (22% prevalence) significantly increased weaning failure risk (27). This observation underscores the critical importance of considering underlying respiratory pathology in weaning strategies. In our cohort, patients with multiple comorbidities showed progressively worse outcomes, particularly those with both COPD and cardiovascular disease (38.7% failure rate, 5.2 ± 1.9 days ventilation duration). This aligns with WEAN SAFE data showing hospital mortality increasing from 16% (no comorbidity) to 34% (≥ 3 comorbidities) (0). The synergistic negative effect of multiple comorbidities on weaning outcomes emphasizes the need for more careful assessment and individualized approaches in these high-risk patients. Our findings on diaphragmatic function echo recent evidence that diaphragm thickness (threshold ≥ 2 mm) significantly impacts weaning predictor accuracy (31), supporting the need for population-specific assessment approaches. This relationship between diaphragm parameters and weaning outcomes varies across different patient subgroups, suggesting that weaning protocols may need to be tailored based on specific patient characteristics. Age emerged as an important factor in our analysis, with older patients showing increased weaning difficulty. This aligns with recent evidence that both frailty (CFS > 4) and advanced age (≥ 80 years) independently predict weaning outcomes (32). However, our data suggests that underlying comorbidities may have a more significant impact than age alone on weaning success. Regarding post-extubation support strategies, while prophylactic NIV has been shown to reduce reintubation (OR: 0.49; 95% CI: 0.32–0.74) and ICU mortality (OR: 0.39; 95% CI: 0.21–0.71) (33), our subgroup analysis suggests intervention effectiveness varies by underlying pathology and comorbidity profile. This highlights the importance of selecting appropriate post-extubation support strategies based on individual patient characteristics.

The main strengths of this study include the introduction of a novel and dynamic weaning predictor, the rigorous methodology, and the comprehensive analysis of multiple weaning parameters. The ΔDE_30–0_ ratio offers a unique perspective on diaphragmatic function by capturing the temporal changes in diaphragm excursion over a 30-min period, which is not addressed by existing indices like DE or DTF (9).

We acknowledge several important limitations in our study. First, the retrospective single-center design (*n* = 100) inherently carries potential selection bias, although we attempted to minimize this by including consecutive patients meeting our criteria. To address this limitation, we recommend implementing standardized measurement protocols across multiple centers to enhance reproducibility and external validity. While we excluded patients with neuromuscular disorders and chest wall deformities, we recognize that several important confounding factors were not fully controlled, including cumulative sedative doses and sedation strategies (34, 35), nutritional status and muscle mass preservation (36), prior duration of mechanical ventilation (37), and variations in weaning protocols among different attending physicians (38). Second, our data showed a predominance of patients with moderate disease severity (mean APACHE II: 21.55 ± 5.98), which reflects our institution’s typical patient population but may affect generalizability. Third, as a single-center study, our findings reflect specific institutional protocols and management strategies, which may vary across different healthcare settings. Additionally, the measurement of diaphragm excursion using ultrasound is operator-dependent and may introduce variability (7, 33). To minimize technical variability, we propose regular operator training and competency assessments, along with the development of automated measurement systems.

For successful clinical integration of ΔDE_30–0_ into routine practice, we propose a comprehensive implementation framework. First, standardized operating procedures with clear measurement guidelines should be developed and disseminated to ensure consistent assessment techniques across different operators and settings. These procedures should be integrated into electronic health records systems, enabling real-time decision support and automated documentation of measurements. To maintain measurement accuracy, regular calibration of ultrasound equipment must be performed according to manufacturer specifications, complemented by rigorous quality control measures including periodic inter-observer reliability assessments. Additionally, healthcare teams should establish and follow rapid response protocols when patients exhibit adverse changes in ΔDE_30–0_ values, allowing for timely intervention and adjustment of weaning strategies. This systematic approach to clinical implementation would help maximize the utility of ΔDE_30–0_ as a weaning predictor while ensuring measurement reliability and patient safety.

While these limitations warrant consideration, we believe the robust association between ΔDE_30–0_ and weaning outcomes observed in our study provides a strong foundation for future multicenter validation studies. The implementation of these proposed strategies and recommendations would significantly enhance the reliability and clinical utility of ΔDE_30–0_ as a weaning predictor.

Looking ahead, several critical research directions emerge from our findings. First, large-scale multicenter validation studies are essential to confirm the predictive value of ΔDE_30–0_ across diverse patient populations and institutional settings. These studies should implement standardized protocols for sedation management following the latest ICU guidelines (39), nutritional support based on ESPEN recommendations (40, 41), and weaning procedures aligned with ACCP/ATS clinical practice guidelines (42). Second, investigating the relationship between ΔDE_30–0_ and long-term outcomes, including post-extubation quality of life and functional status, would provide valuable insights into the clinical utility of this index. This research should include comprehensive follow-up studies to understand the association between ventilation durations, weaning patterns, and patient outcomes across various disease profiles. Third, the development and validation of machine learning algorithms incorporating ΔDE_30–0_ for automated weaning prediction represents an innovative direction. These algorithms could integrate multiple clinical, physiological, and biochemical parameters to create more sophisticated prediction models. To ensure reliability, we recommend establishing comprehensive ultrasound training programs following international competency standards (43) to minimize operator-dependent variability. Fourth, cost-effectiveness analysis of routine ΔDE_30–0_ monitoring in clinical practice is crucial. This analysis should evaluate the economic impact of implementing this technique, including resources required for training, equipment, and personnel time. Additionally, assessment of the impact on healthcare resource utilization and ICU length of stay would provide valuable information for healthcare systems considering widespread adoption. Finally, studies should aim to establish optimal cut-off values and evaluate the performance of this novel index in different patient subgroups, particularly those with pre-existing respiratory comorbidities or specific etiologies of respiratory failure (44). The implementation of these standardized protocols and multicenter validation would significantly strengthen the evidence base for using ΔDE_30–0_ as a reliable predictor of weaning success, potentially improving patient outcomes and resource allocation in the ICU (45).

In conclusion, the 30-min diaphragm excursion change rate (ΔDE_30–0_) demonstrates promising potential as a novel predictor of weaning success in patients with severe pneumonia requiring invasive mechanical ventilation. The superior predictive performance of this index compared to existing weaning parameters highlights its clinical relevance and potential impact on improving weaning outcomes. Future research should focus on prospective validation, establishment of optimal cut-off values, and the development of integrated clinical prediction tools to enhance the accuracy and reliability of weaning decision-making in the ICU.

## 5 Conclusion

In this study, we introduced the 30-min diaphragm excursion change rate (ΔDE_30–0_) as a novel predictor of weaning success in patients with severe pneumonia requiring invasive mechanical ventilation. The main findings demonstrate that the ΔDE_30–0_ ratio had the highest predictive performance among the evaluated weaning parameters, with an AUROC of 0.924, a sensitivity of 86.100%, and a specificity of 95.200% at a cut-off value of 0.209. These results highlight the potential of this dynamic index in improving the accuracy and reliability of weaning assessments in critically ill patients. However, while these initial findings are promising, we acknowledge that larger multicenter validation studies are essential to confirm the generalizability of our results across diverse patient populations and clinical settings. Furthermore, future research should focus on standardizing measurement protocols and establishing the role of ΔDE_30–0_ in different pathological conditions before its widespread implementation in clinical practice.

## Data Availability

The original contributions presented in the study are included in the article/supplementary material, further inquiries can be directed to the corresponding author.
